# Drs. Hall & Gibbs

**Published:** 1875-10

**Authors:** 


### DRS. HALL & GIBBS.

Those of our readers who are so unfortunate as to need medical advice will not find it amiss to observe the fact that Dr. W. W. HALL and Dr. E. H. GIBBS have united their forces, and are to be found daily at their rooms, No. 137 Eighth Street, New York. Those who have for years listened to their teachings in the pages of this *Journal*, need not be told that while their province is to heal the sick, they are not great admirers of copious drugging, but in other and better agencies. Much of their work is done by correspondence, and this they invite.

---

HALL'S JOURNAL OF HEALTH for 1874, neatly bound in red cloth, nearly 500 pages, will be sent free to any address on receipt of $1.25, being only one-half the regular price. Only a few copies remain.

---

### THE INSTITUTE FAIR.

It will be observed that we devote a good deal of our space to descriptions of articles on exhibition at the great Fair of the American Institute, which is now in the full tide of success. The exhibition is a grand one, comprising much of interest to all classes. The Art department is very full; and the display of new and useful machines and inventions is unusually complete. As the prophet and harbinger of the grand Centennial event, it may be deemed a great success,—foreshadowing, as it does, an approaching grandeur of display in all that is useful and attractive, such as has never yet been excelled in any clime or by any people. Our citizens, as well as strangers, should not allow this American Institute Fair to close without one visit or more, of careful and prolonged inspection.

